# Phylotocol: Promoting Transparency and Overcoming Bias in Phylogenetics

**DOI:** 10.1093/sysbio/syy090

**Published:** 2018-12-31

**Authors:** Melissa B DeBiasse, Joseph F Ryan

**Affiliations:** 1 Whitney Laboratory for Marine Bioscience, 9505 Ocean Shore Boulevard, St. Augustine, FL 32080, USA; 2 Department of Biology, University of Florida, 220 Bartram Hall, Gainesville, FL, 32611, USA

**Keywords:** Accountability, confirmation bias, open science, phylogenetics, phylotocol, protocol, transparency

## Abstract

The integrity of science requires that the process be based on sound experimental design and objective methodology. Strategies that increase reproducibility and transparency in science protect this integrity by reducing conscious and unconscious biases. Given the large number of analysis options and the constant development of new methodologies in phylogenetics, this field is one that would particularly benefit from more transparent research design. Herein, we introduce phylotocol (fi lō ’ta kôl), an *a priori* protocol-driven approach in which all analyses are planned and documented at the start of a project. The phylotocol template is simple and the implementation options are flexible to reduce administrative burdens and allow researchers to adapt it to their needs without restricting scientific creativity. While the primary goal of phylotocol is to increase transparency and accountability, it has a number of auxiliary benefits including improving study design and reproducibility, enhancing collaboration and education, and increasing the likelihood of project completion. Our goal with this *Point of View* article is to encourage a dialog about transparency in phylogenetics and the best strategies to bring transparent research practices to our field.

The production of reliable scientific results depends upon objective methodology. Reproducibility and transparency are safeguards against conscious and unconscious biases in scientific inquiry. The importance of reproducibility in science has been written about extensively over the past decade (King et al. 2009; McNutt 2014; Markowetz 2015), but its counterpart, transparency, has only recently begun to receive serious consideration (Ihle et al. 2017; Nosek et al. 2018). A reproducible study contains methods required to replicate all *reported* results, but it does not necessarily include all decisions that led to the final methodology reported in a study. Therefore, a reproducible study is not necessarily a transparent one. For example, if researchers present only a subset of results along with the methods required to generate those results (reporting bias), the study is technically reproducible, but lacks transparency. This lack of transparency is a problem across scientific disciplines, and is particularly applicable to phylogenetics.

Inferring relationships between genes, genomes, and species is essential for a fundamental understanding of biology. In the nearly 70 years since Hennig formalized phylogenetics (Hennig 1965), the field has matured through the continuous development and improvement of algorithms, models, and data manipulation strategies (Whelan et al. 2001) leading to many advances in phylogenetic methodology. However, the continual nature of methodological improvement and growing number of analysis options impedes standardization of experimental design. While as scientists we strive for objectivity and impartiality, we are all susceptible to conscious and unconscious biases (Kunda 1990; Christensen and Willham 1991; Nickerson 1998; Pronin and Kugler 2007; Nosek and Riskind 2012), and implementing strategies to reduce the influence of these biases in our experiments is important for the integrity of science.

For most phylogenetic analyses, phylogeneticists are faced with a seemingly infinite combination of algorithms, models, and data manipulation techniques. Some examples related to tree reconstruction include: algorithms [e.g., distance, parsimony, maximum likelihood, and Bayesian inference (Felsenstein 2004)], single-matrix models (e.g., JTT and WAG), criteria to determine model fit [e.g., AIC and BIC (Page and Holmes 2009)], partitioning and mixture model schemes (Blair and Murphy 2010), data filtering [e.g., removing unstable and quickly evolving taxa or genes (Salichos and Rokas 2013)]. Other phylogenetic applications (e.g., molecular clock analyses, ancestral state reconstruction, hypothesis testing, and detection of selection) require researchers to make comparable decisions between competing approaches (Baum and Smith 2013).

In phylogenetics, research plans are generally informal and rarely outlined in detail prior to the start of the project; rather, plans are often constructed gradually, with each next step motivated by the results of the step before, an approach Gelman and Loken (2014) refer to as the “garden of forking paths.” This strategy is problematic because the selection of some paths is more likely than the selection of others, particularly if researchers make downstream methodological decisions consciously or, more often, unconsciously, in response to results that conflict with expected outcomes. Statistically the garden of forking paths is a problem because it makes correcting $P$-values for multiple comparisons impossible, rending them uninterpretable (Tukey 1949; Dunnett 1955).

In clinical trials, where the outcomes of a study can put human lives at risk, biases have been explicitly controlled for, and transparency and reproducibility ensured, through the requirement of *a priori* protocols that outline objective(s), design, methodology, statistical considerations, and study organization (Laine et al. 2007; Zarin and Tse 2013; Zarin et al. 2017). Protocols must be registered to a governmental regulatory agency, funding agency, and/or an institutional review board prior to the start of a study. Any changes (amendments) to a protocol require explicit justification and an updated version of the protocol (Getz et al. 2016). Many journals require protocols to be published with clinical trial publications, providing further motivation for their implementation. After the creation of the ClinicalTrials.gov registry (USFDA 1997) led to the widespread adoption of transparent reporting standards in clinical trials, a dramatic drop in the frequency of positive results was observed, suggesting that bias may have been inflating the number positive results (Kaplan and Irvin 2015). Protocols greatly reduce, if not eliminate, the potential for researcher bias and in the process ensure the safety of subjects and the integrity of the trial.

Recently, *a priori* analysis plans and the preregistration of research designs have been proposed to promote transparency in the fields of Behavioral Ecology (Ihle et al. 2017), Ecology and Evolution (Parker et al. 2016), and Psychology (Hartgerink and Wicherts 2016) and a multidisciplinary working group has established a framework for minimal reporting standards (Aalbersberg et al. 2018). The proposed measures are comparable to protocol registration in clinical trials and provide effective means to promote transparency in each particular field. Responses to these efforts have been positive (Blumstein 2017; Forstmeier 2017; Parker and Nakagawa 2017), negative (Koenig 2017), and mixed (Cockburn 2017; Hatchwell 2017). The biggest barrier to widespread adoption to preregistration is the administrative effort associated with its implementation, perceived restrictions on scientific creativity and exploratory analyses, and concerns that project ideas will be scooped.

We argue that the field of phylogenetics would benefit tremendously from increased transparency. Herein, we introduce phylotocol, an *a priori* protocol-driven approach in which all analyses are planned and documented at the inception of a project, and optionally are preregistered. Phylotocol can be easily incorporated into phylogenetic studies; we have been using phylotocol since June 2017 and find it improves the rigor and efficiency of our research generally and our experimental design specifically. Herein, we describe a phylotocol template in detail, propose a set of guidelines for its use, include examples of phylotocols that we have implemented in our own research, and discuss how using a phylotocol can reduce bias and improve transparency and reproducibility in phylogenetics with minimal burdens on researchers’ time. Our goal is to start a dialog about the importance of transparency in phylogenetics and suggest ways to increase transparency and accountability in the field.

## Anatomy of Phylotocol

**Figure 1.**
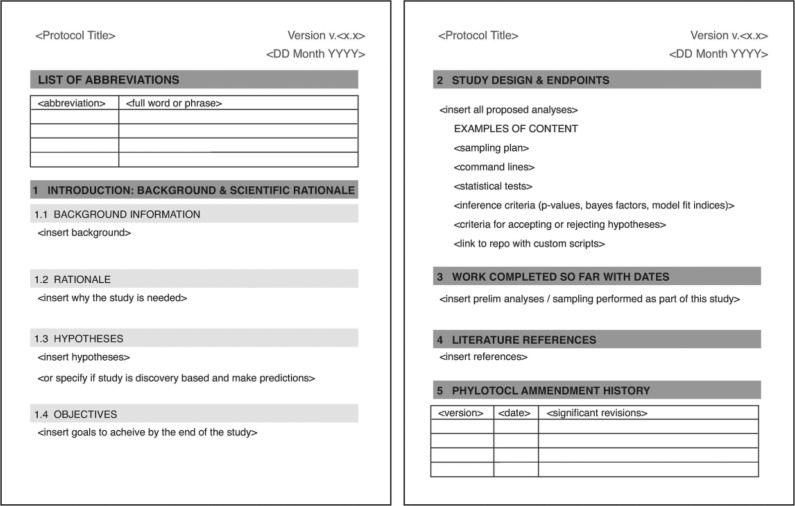
Phylotocol template. Based on the NIH clinical trial protocol, the phylotocol layout has been tailored to match the needs of phylogenetic research. The format and the information required are flexible. The figure displays the Microsoft Word version of the template, but there is also a markdown version. A phylotocol can be used as the basis for preregistration, uploaded to any online repository, or kept as a personal document (see Implementation section).

The template phylotocol is based on the clinical trial protocol established by the National Institutes of Health (Hudson et al. 2016) and has seven major sections: (1) Title, (2) Abbreviations, (3) Introduction, (4) Study design, (5) Steps completed, (6) References, and (7) Appendix with version history (Fig. 1). This minimalist format reduces unnecessary burden, lowering the bar for implementation, but is flexible and can be customized to the requirements, preference, and computational expertise of a particular user. As opposed to a detailed template that might stifle creativity, the minimalist strategy is intended to foster the emergence of best practices, which we anticipate will evolve over time. Blank phylotocol templates in Microsoft Word and markdown formats and publicly posted phylotocols for research projects in the Ryan Lab are available at the following link (https://github.com/josephryan/phylotocol) and in the Supplementary Materials (Online Appendices 1–5 available on Dryad at http://dx.doi.org/10.5061/dryad.n2q57qn).

A phylotocol is an outline of all decisions that could affect the final outcome of a study. Some common decisions include: (1) central hypotheses, (2) how taxa and data will be filtered, (3) which methods will be applied, (4) which models will be implemented, and (5) which criteria will be used to validate or reject hypotheses. While not required, we recommend including command lines and parameter settings (e.g., number of starting trees, seeds used for programs with random processes, minimum occupancy of phylogenomic matrices) to maximize clarity. Writing a phylotocol forces researchers to anticipate difficult decisions; for example, when applying different algorithms, models, etc. to the same data matrix, it is important to provide explicit criteria for evaluating conflicting results.

Ideally, a researcher would plan all steps in an analysis pipeline before testing is started, but in many cases, adjustments to the plan are needed once experiments are underway. The appendix section of the phylotocol is designed to accommodate changes to the analysis pipeline, for example, including an improved method that has recently been released, adding newly available data to a study, adjusting parameter settings, or correcting obvious mistakes. Each change should be accompanied by a justification and documentation of work completed so far, the latter making it possible to determine at which stage of a project a change was made.

## Primary Objectives of Phylotocol

The primary objective of phylotocol is to increase transparency and accountability in phylogenetics. By outlining analyses *a priori*, phylotocol promotes transparency and reduces biases on the part of researchers. While many decisions made during the course of a study are obviously free of bias, and others clearly driven by bias are avoided by the majority of researchers, most decisions fall somewhere along this spectrum. By integrating transparency into a study, researchers provide readers with the ability to evaluate the validity of these decisions.

A transparent study reports all steps in the pipeline, even those that were replaced by other methods, or those that motivated downstream analyses but were not explicitly addressed in the final manuscript. In this way, phylotocol differs from traditional methods or supplementary methods sections, which typically only describe methodology for results that are reported in a manuscript. As is the case with Supplementary Materials available on Dryad, it is likely that a casual reader of the study will not be interested in the technical details supplied in a phylotocol; however, these details will be extremely important to researchers who are replicating or building upon the results of the study.

Accountability is a natural by-product of transparency (Mellor et al. 2018). In phylogenetics, as in other fields, it can be tempting to modify analyses when results conflict with our expectations. By implementing a phylotocol, researchers acknowledge that they are accountable for changes made during the period of a study and will be more motivated to deeply consider the implications of post-hoc decisions on the outcome of the analyses and the interpretation of the results.

## Auxiliary Benefits of Phylotocol

While the primary goal of phylotocol is to increase transparency and accountability, the process offers a number of auxiliary benefits, which we describe below.

### Designing a Better Study

Outlining each step of a study in a phylotocol before analyses are started can bring about a more robust plan. The process of transcribing procedures and guidelines for the interpretation of results can identify important steps and logical flaws that might otherwise be overlooked in a more patchwork experimental design. Catching these obstacles early in the process can lead to huge savings in time and/or money.

### Documentation

Unlike in wet-lab based experimental biology, keeping a formal notebook to record the details of an analysis is less commonplace in phylogenetics. Creating a phylotocol that is updated throughout duration of a project helps serve many of the same purposes of a lab notebook. In this manner, a phylotocol serves as a key reference document for constructing the methods section of a manuscript.

### Collaboration

Creating and executing a phylotocol can facilitate seamless collaborations among research groups. Getting input early from collaborators can strengthen a study while also ensuring that effort between collaborators does not overlap. Listing all steps also allows computational, personnel, budgetary, and other resource needs to be assessed. When collaborators agree on the analyses before a project is initiated, it helps prevent misunderstandings and/or conflicts down the line.

### Education

Phylotocol provides an excellent framework from which to train early career scientists. During the process of constructing a phylotocol, students gain a deeper understanding of the components of the study. Later, they have a roadmap from which to work throughout the project and mentors can be sure that effort is focused appropriately. Furthermore, previous phylotocols are useful references for new lab members who want to quickly get up to speed on how the lab performs particular analyses and can act as a template from which to start new analyses. Phylotocols can easily be incorporated into undergraduate and graduate courses as a tool to teach methodology, the importance of robust experimental design, and to reinforce the concepts of transparency and reproducibility in science.

### Project Completion

The inherent open-endedness of science can often be intimidating and create a barrier to project completion. Implementing a phylotocol can remove this barrier by providing explicit starting and stopping points for a project and the motivation to complete the study as planned. The phylotocol quantifies the number of objectives a project requires and helps researchers prioritize each step. Beginning and completing a manuscript for the project will also be less daunting because the background information, study justification, methods, and references will already be compiled in the phylotocol. Starting new projects hinders the ability to complete existing projects; a phylotocol serves as a gentle impediment to spontaneously starting tangential projects and therefore increases productivity.

## Implementation of Phylotocol

**Figure 2.**
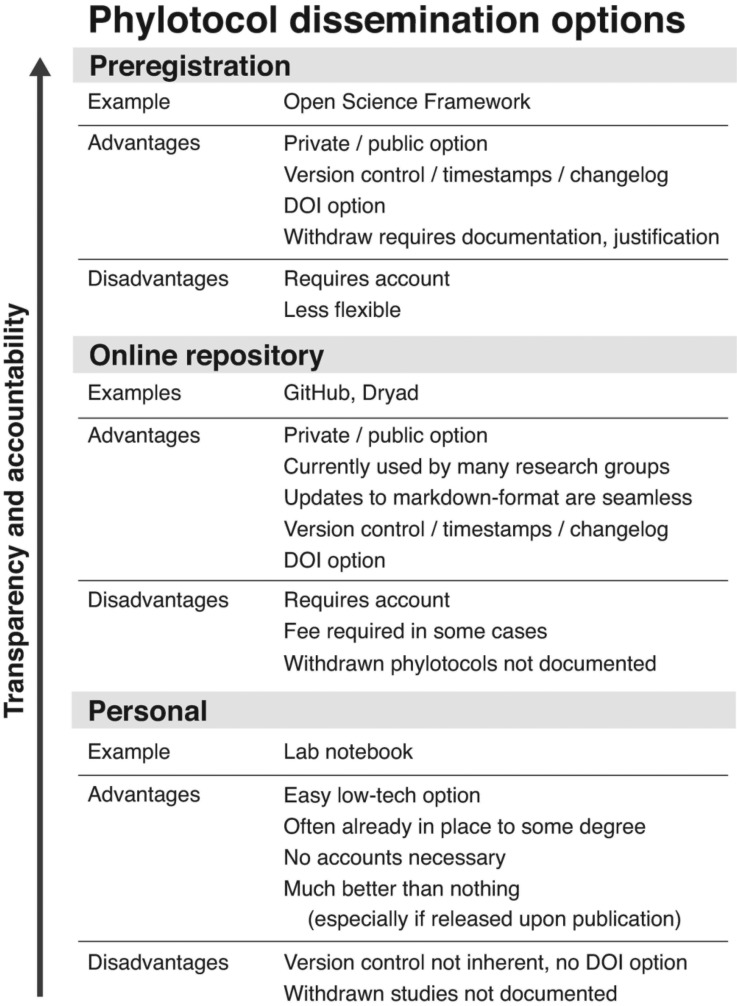
Implementation options. There are three frameworks for implementing a phylotocol, each with increasing returns on transparency and accountability, as indicated by the arrow. For each framework, an example strategy is listed with its associated advantages and disadvantages. The preregistration framework provides a superior level of transparency, but the repository and personal frameworks still provide benefits and are especially useful for getting started with phylotocol.

There are several ways to implement phylotocol (Fig. 2). The option with the highest returns on transparency and accountability is preregistration with an organization such as the Open Science Framework (OSF, https://osf.io/) (Nosek et al. 2015). If using OSF to post a phylotocol, we recommend choosing the “Open-Ended Registration” option and pasting a text version of phylotocol into the box. The OSF registry has an embargo system which keeps a registration private for up to 4 years, but ensures that a preregistered study is eventually released, whether published or not. A preregistration can be withdrawn but the title is still released and a justification is required. OSF also allows users to connect registrations to workflow management tools (e.g., Dataverse, Dropbox, figshare, Github, and others, see: http://help.osf.io/m/addons), so that contributions from different members of a research team can be connected, persistently stored, and cited in one location. When researchers are ready to disseminate early findings, any file on the OSF can be given a digital object identifier (DOI) and shared as a preprint (https://osf.io/preprints) prior to publication in a journal. One drawback to posting a phylotocol on OSF is that the original document cannot be edited. If changes to the phylotocol are needed, a new version must be uploaded. Another small drawback is that posting to OSF requires registering for an account and keeping track of credentials.

A second way to implement phylotocol is to post the document to an online software or data repository, such as GitHub or Dryad. Many users have experience with one or more of these repositories, so the learning curve with this option is minimal. The specific features of different online repositories vary, but most have a timestamp feature to provide transparency as to when a phylotocol is posted and edited, version control, which allows for seamless updating (especially when implementing a markdown version of phylotocol), and DOI assignment. Most repositories allow documents to remain private, but a drawback in terms of promoting transparency is that if a study is discontinued or substantially changed, there is no requirement to release the phylotocol or justify the retraction. This could present a transparency problem if a future work relies on data generated as part of an unfinished study. Like preregistration, online repositories also have the minor inconvenience of requiring users to create an account and keep track of credentials.

The third way to implement a phylotocol is to create a private document on a personal computer or in a lab notebook. This is the most simple, low tech, and flexible option and does not require making an account or remembering a password. This strategy lacks the built-in version control and timestamp features of the above options, which is a disadvantage (although version control software can be implemented secondarily). In addition, like a phylotocol privately posted to an online repository, there is no requirement that a document kept in a lab notebook be made public, limiting the transparency of the process. However, this strategy can greatly increase the transparency of a project and researchers who choose this option will greatly benefit from implementing a personal phylotocol.

The multiple flexible options for implementing phylotocol, each with various levels of commitment, make it easy to try out the process. Researchers interested in incorporating more transparent practices in their research could ease into phylotocol by first making private documents for their own use. Once familiar with the process, they can transition to posting the phylotocol to an online repository, and then move toward preregistration, which is the gold standard for transparency and accountability. Each step along this progression requires a higher level of commitment, but we predict that the structure will serve many researchers well.

## Discussion

The production of reliable and bias-free results is an indisputable goal of all phylogenetic studies. By planning analyses before a study begins, and making methodological choices transparent, a phylotocol reduces the likelihood of confirming a false hypothesis. A phylotocol, therefore, makes considerable contribution toward reaching the goal of strong, bias-free research results.

The idea of including additional steps to an already time-consuming research process will almost certainly be met with hesitation, if not objection, but we contend that the time spent on phylotocol is easily recovered both in the short and long term. In practice, we have found that time invested in phylotocol pays dividends downstream, particularly when training junior researchers, writing manuscripts, and keeping projects on track toward completion. In the long run, wide adoption of phylotocol will lead to less confirmation bias in the scientific record and therefore huge savings in time that would otherwise be spent building upon or rebutting questionable results.

A major concern is that implementing a phylotocol will stifle scientific creativity and data exploration (Koenig 2017). We contend that phylotocol and creative data exploration are not mutually exclusive, and that in some ways, phylotocol enhances the creative process. Writing a phylotocol explicitly requires that researchers dedicate time to planning a study start to finish, which can be an inherently creative process, potentially more so than planning the analyses haphazardly or informally. Furthermore, phylotocol does include built-in support for unplanned exploratory analyses through appendix updates. Decisions to add, change, or disregard planned analyses require only that changes be documented and justified.

As scientists, our ultimate goal is to make discoveries and formulate theories that stand up to rigorous testing, and eventually become widely accepted as truth. The possibility that bias can inadvertently influence our research results should not be minimized or neglected. By implementing phylotocol phylogeneticists will show dedication to scientific integrity, which will lead to confidence in the reliability of their work. In this way, transparent research practices like phylotocol help maximize research impact.

## Conclusions

Phylotocol is a powerful tool to increase transparency and accountability in phylogenetics. It has great potential to improve how phylogenetic research is conducted, interpreted, communicated, and perceived. The implementation is straightforward and offers a range of auxiliary benefits, including making contributions to study design, reproducibility, collaboration, and education. Phylotocol can bolster scientific productivity both at the level of the individual researcher as well as in the broader context of the scientific record. While phylotocol is a simple idea, its repercussions could be far reaching if widely implemented.

## Supplementary Material

Data available from the Dryad Digital Repository: http://dx.doi.org/10.5061/dryad.n2q57qn
